# Supplementary material for: Reducing Antimicrobial Usage in Small-Scale Chicken Farms in Vietnam: A 3-Year Intervention Study
Source: Front Vet Sci. 2021 Jan 28;7:612993. doi: 10.3389/fvets.2020.612993 (PMC7876082; doi:10.3389/fvets.2020.612993)
Supplement: Supplementary Table 1 — Descriptive characteristics of chicken farms by total of farms, flocks, and weeks. [file Table_3.docx]

**Supplementary Table 3.** Descriptive characteristics of chicken farms by total of farms, flocks and weeks.

| Demographic and chicken farming information |  | Baseline of  all farms | | |  | Baseline of  intervention farms | | |  | Transition | | |  | Intervention | | |  | Control | | |
| --- | --- | --- | --- | --- | --- | --- | --- | --- | --- | --- | --- | --- | --- | --- | --- | --- | --- | --- | --- | --- |
|  |  | Farms  (102) | Flocks (219) | Weeks  (3,899) |  | Farms  (31) | Flocks  (87) | Weeks  (1,551) |  | Farms  (18) | Flocks  (22) | Weeks  (396) |  | Farms  (31) | Flocks  (77) | Weeks  (1,350) |  | Farms  (4) | Flocks  (12) | Weeks  (227) |
| Farmer’s gender |  |  |  |  |  |  |  |  |  |  |  |  |  |  |  |  |  |  |  |  |
| *Female* |  | 12 | 35 | 611 |  | 4 | 15 | 273 |  | 3 | 4 | 68 |  | 4 | 9 | 150 |  | 1 | 3 | 65 |
| *Male* |  | 90 | 184 | 3,288 |  | 27 | 72 | 1,278 |  | 15 | 18 | 328 |  | 27 | 68 | 1,200 |  | 3 | 9 | 162 |
| Farmer’s age |  |  |  |  |  |  |  |  |  |  |  |  |  |  |  |  |  |  |  |  |
| *<=35* |  | 19 | 40 | 677 |  | 4 | 9 | 145 |  | 4 | 4 | 77 |  | 4 | 7 | 138 |  | 0 | 0 | 0 |
| *36-45* |  | 30 | 61 | 1,055 |  | 7 | 22 | 374 |  | 7 | 11 | 188 |  | 7 | 23 | 372 |  | 0 | 0 | 0 |
| *46-55* |  | 29 | 60 | 1,108 |  | 9 | 27 | 507 |  | 3 | 3 | 58 |  | 9 | 19 | 336 |  | 2 | 6 | 99 |
| *>55* |  | 24 | 58 | 1,059 |  | 11 | 29 | 525 |  | 4 | 4 | 73 |  | 11 | 28 | 504 |  | 2 | 6 | 128 |
| District (%) |  |  |  |  |  |  |  |  |  |  |  |  |  |  |  |  |  |  |  |  |
| *Cao Lanh* |  | 48 | 96 | 1,667 |  | 17 | 46 | 808 |  | 7 | 10 | 172 |  | 17 | 47 | 822 |  | 1 | 5 | 84 |
| *Thap Muoi* |  | 54 | 123 | 2,232 |  | 14 | 41 | 743 |  | 11 | 12 | 224 |  | 14 | 30 | 528 |  | 3 | 7 | 143 |
| Education status |  |  |  |  |  |  |  |  |  |  |  |  |  |  |  |  |  |  |  |  |
| *Primary school* |  | 26 | 62 | 1,133 |  | 13 | 40 | 732 |  | 7 | 9 | 167 |  | 13 | 39 | 699 |  | 0 | 0 | 0 |
| *Secondary school* |  | 42 | 91 | 1,585 |  | 11 | 25 | 418 |  | 5 | 6 | 101 |  | 11 | 28 | 468 |  | 4 | 12 | 227 |
| *High school* |  | 29 | 54 | 942 |  | 5 | 14 | 241 |  | 5 | 5 | 93 |  | 5 | 6 | 111 |  | 0 | 0 | 0 |
| *Post high school* |  | 5 | 12 | 239 |  | 2 | 8 | 160 |  | 1 | 2 | 35 |  | 2 | 4 | 72 |  | 0 | 0 | 0 |
| Chicken farming experience in years |  |  |  |  |  |  |  |  |  |  |  |  |  |  |  |  |  |  |  |  |
| *0-1* |  | 23 | 52 | 972 |  | 8 | 28 | 512 |  | 7 | 9 | 159 |  | 8 | 25 | 448 |  | 0 | 0 | 0 |
| *>1-2* |  | 37 | 80 | 1,364 |  | 14 | 32 | 556 |  | 5 | 6 | 101 |  | 14 | 32 | 538 |  | 1 | 3 | 63 |
| *>2-4* |  | 29 | 59 | 1,060 |  | 6 | 17 | 307 |  | 3 | 3 | 62 |  | 6 | 16 | 286 |  | 3 | 9 | 164 |
| *>4* |  | 13 | 28 | 503 |  | 3 | 10 | 176 |  | 3 | 4 | 74 |  | 3 | 4 | 78 |  | 0 | 0 | 0 |
| Number of restocked chicks |  |  |  |  |  |  |  |  |  |  |  |  |  |  |  |  |  |  |  |  |
| *100-199* |  | 27 | 41 | 670 |  | 9 | 19 | 308 |  | 4 | 5 | 81 |  | 7 | 22 | 369 |  | 1 | 1 | 15 |
| *200-299* |  | 36 | 60 | 1,044 |  | 9 | 18 | 324 |  | 5 | 5 | 94 |  | 9 | 11 | 184 |  | 1 | 2 | 44 |
| *300-499* |  | 45 | 62 | 1,109 |  | 14 | 20 | 351 |  | 3 | 3 | 51 |  | 11 | 20 | 344 |  | 3 | 3 | 57 |
| *>500* |  | 29 | 56 | 1,076 |  | 14 | 30 | 568 |  | 7 | 9 | 170 |  | 12 | 24 | 453 |  | 2 | 6 | 111 |
| Week of production cycle |  |  |  |  |  |  |  |  |  |  |  |  |  |  |  |  |  |  |  |  |
| *1-5* |  | 1 | 2 | 10 |  | 0 | 0 | 0 |  | 0 | 0 | 0 |  | 0 | 0 | 0 |  | 0 | 0 | 0 |
| *6-10* |  | 10 | 10 | 91 |  | 2 | 2 | 18 |  | 0 | 0 | 0 |  | 2 | 2 | 19 |  | 0 | 0 | 0 |
| *11-15* |  | 24 | 29 | 418 |  | 8 | 10 | 142 |  | 3 | 3 | 45 |  | 11 | 13 | 190 |  | 2 | 2 | 29 |
| *16-20* |  | 81 | 136 | 2,442 |  | 31 | 62 | 1,102 |  | 15 | 17 | 307 |  | 26 | 53 | 939 |  | 2 | 6 | 108 |
| *>20* |  | 32 | 42 | 938 |  | 8 | 13 | 289 |  | 2 | 2 | 44 |  | 8 | 9 | 202 |  | 2 | 4 | 90 |
| Presence of other chicken flocks |  |  |  |  |  |  |  |  |  |  |  |  |  |  |  |  |  |  |  |  |
| *Yes* |  | 77 | 145 | 2,617 |  | 25 | 56 | 1,020 |  | 12 | 16 | 290 |  | 22 | 51 | 881 |  | 4 | 9 | 176 |
| *No* |  | 52 | 74 | 1,282 |  | 19 | 31 | 531 |  | 6 | 6 | 106 |  | 19 | 26 | 469 |  | 2 | 3 | 51 |
| Presence of non-chicken species |  |  |  |  |  |  |  |  |  |  |  |  |  |  |  |  |  |  |  |  |
| *Yes* |  | 93 | 194 | 3,435 |  | 31 | 84 | 1,493 |  | 17 | 20 | 365 |  | 31 | 75 | 1,312 |  | 4 | 12 | 227 |
| *No* |  | 16 | 25 | 464 |  | 2 | 3 | 58 |  | 2 | 2 | 31 |  | 1 | 2 | 38 |  | 0 | 0 | 0 |
| Sources of chicks |  |  |  |  |  |  |  |  |  |  |  |  |  |  |  |  |  |  |  |  |
| *Company hatchery* |  | 2 | 2 | 41 |  | 0 | 0 | 0 |  | 0 | 0 | 0 |  | 0 | 0 | 0 |  | 0 | 0 | 0 |
| *Local hatchery* |  | 38 | 62 | 1,147 |  | 11 | 25 | 450 |  | 5 | 7 | 125 |  | 11 | 28 | 521 |  | 2 | 6 | 112 |
| *Own farm hatchery* |  | 1 | 1 | 17 |  | 1 | 1 | 17 |  | 0 | 0 | 0 |  | 1 | 1 | 17 |  | 0 | 0 | 0 |
| *Dealer* |  | 76 | 154 | 2,694 |  | 25 | 61 | 1,084 |  | 13 | 15 | 271 |  | 21 | 48 | 812 |  | 4 | 6 | 115 |
